# Hydrogen-rich saline ameliorates the retina against light-induced damage in rats

**DOI:** 10.1186/2045-9912-3-19

**Published:** 2013-09-01

**Authors:** Liang Tian, Lei Zhang, Feng Xia, Jing An, Yashino Sugita, Zuoming Zhang

**Affiliations:** 1 Department of Clinical Medicine, Faculty of Aerospace Medicine, Key Laboratory of Aerospace Medicine of the National Education Ministry, Fourth Military University, 17 Changle West Road, Xi’an 710032, China

**Keywords:** Light damage, Hydrogen, Retina

## Abstract

Oxidative reactions are thought to be a major cause of light-induced retinal degeneration. This study was designed to investigate the effects of hydrogen-rich saline (HRS) on the prevention and treatment of light-induced retinal injury in rats. Male Sprague–Dawley rats were divided randomly into three groups: light damage, HRS prevention (5 ml/kg, 30 min before intensive light exposure), and HRS treatment (5 ml/kg per day for 5 days, after intensive light exposure), respectively. The right eye of each rat was exposed to 5000 lux constant white light-emitting diode (LED) light for 3 h, and the left eye was covered to serve as the blank control. Electroretinograms were recorded 5 days later, and the thickness of the outer nuclear layer (ONL) was measured after hematoxylin and eosin (H&E) staining. The results showed that the electroretinogram b-wave amplitudes and the mean ONL thicknesses of rats were significantly greater in the HRS prevention (P < 0.001) and treatment (P < 0.001) groups than in the light damage. These results indicated that peritoneal injection of HRS provides protection and treatment against light-induced retinal degeneration in rats.

## Introduction

Photoreceptors are sensitive to a wide range of light conditions and intensive visible light exposure can lead to photoreceptor degeneration through phototoxic mechanisms [1,2]. Light from the operating microscope used in ophthalmic practice is also a main cause of photic maculopathy [3]. For example, a positive association of cataract surgery and subsequent risk for age-related macular degeneration (AMD) has been reported [4]. Excessive sunlight might also be associated with AMD; therefore, antioxidant nutrients against cell damage might be useful for AMD prevention [5,6]. Natural and synthetic antioxidants used to prevent retinal light damage have been extensively studied [7,8]. For example, retinal damage caused by light exposure can be reduced by various types of antioxidants, such as ascorbate [9], dimethylthiourea [10], thioredoxin [11,12], and NG-nitro-L-arginine-methyl ester (L-NAME) [13,14]. Accordingly, oxidative stress is likely to be involved in the pathogenesis of light-induced retinal damage. But if antioxidants are given after the lights are turned on, the light-induced oxidative damage would be inevitable, implicating that the initial rate of rhodopsin bleaching in the early onset of light exposure is involved in the damage mechanism [7]. However, no antioxidant exhibits complete fidelity with a single species of reactive oxygen and whether an antioxidant can actually pass the blood-retinal barrier and be taken up by the tissue is still controversial. Nevertheless, the effectiveness of a large number of antioxidants is compelling evidence that oxidative stress is an integral part of the light damage process.

Molecular hydrogen has recently been shown to have protective and therapeutic value as an antioxidant through its ability to selectively reduce cytotoxic reactive oxygen species (ROS) [15]. Hydrogen, as a novel antioxidant, has been shown to exert protective effects on transplantation-induced intestinal graft injury [16], chronic liver inflammation [17], vestibular hair cells [18], as well as regional myocardial ischemia and reperfusion [19]. Application of hydrogen-rich saline (HRS, normal saline containing a therapeutic dose of hydrogen) represents an alternative model of delivering molecular hydrogen [17]. The primary advantages of HRS are that it is a portable, easily administered, and safe means of delivering hydrogen [17]. In addition, it has been found that HRS has a protective effect on intestinal ischemia-reperfusion [20], lung injury induced by intestinal ischemia-reperfusion [21], and cerebral ischemia-reperfusion rat models [22]. The protective effect of HRS on small intestine ischemia-reperfusion injury has been revealed possibly by reduction of inflammation and oxidative stress [20]. However, the role that HRS plays in the degeneration of photoreceptor cells during light damage is still unknown, although the effectiveness of HRS has been confirmed in many organs. As a result, the effect of HRS on retinal light damage in rats was investigated in the present study.

## Materials and methods

### Animals

Twenty-four male Sprague–Dawley (SD) rats were obtained from the Laboratory Animal Research Center of the Fourth Military Medical University and were housed with dim (5–10 lux) cyclic light (12 h on/off, 6 AM–6 PM), with food and water available *ad libitum* throughout the studies. Animals (8 weeks of age) were divided randomly into four groups: blank control, light damage, HRS prevention (5 ml/kg, 30 min before intensive light exposure), and HRS treatment (5 ml/kg per day for 5 days, after intensive light exposure), respectively. The right eye exposed to 5000 lux constant white light-emitting diode (LED) light for 3 h was used as the experimental eye, while the left eye was covered and used as the blank control in all rats. All procedures involving animals adhered to the Association for Research in Vision and Ophthalmology (ARVO) Statement for the Use of Animals in Ophthalmic and Vision Research and were approved by the Animal Care and Use Committee of the Fourth Military Medical University.

### Hydrogen-rich saline production

Hydrogen was dissolved in physiological saline for 6 h under high pressure (0.4 MPa) to a supersaturated level using a hydrogen-rich water-producing apparatus produced by our department. The saturated hydrogen saline was stored under atmospheric pressure at 4°C in an aluminum bag with no dead volume. The HRS was sterilized by gamma radiation and was freshly prepared every week, which ensured that a concentration above 0.6 mM was maintained. Gas chromatography was used to confirm the hydrogen content in the saline by the method described by Ohsawa [15].

### Light exposure and HRS treatment

**Figure 1:**
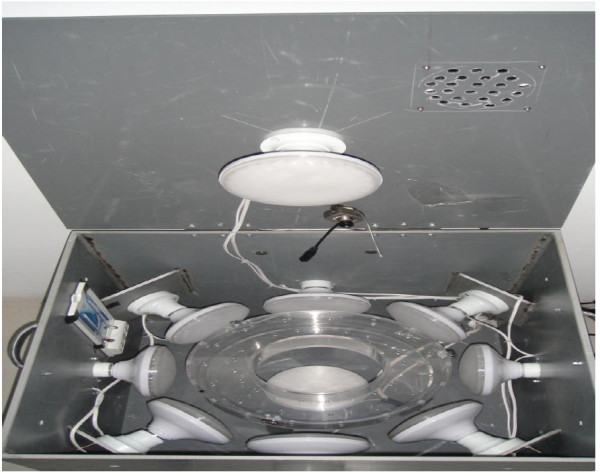
Annular illumination box for light-induced retinal damage in rats.

After adaptation for 12 h in the dark, rats were exposed to intense light randomly for each group. The right eye of rats with natural pupil size was exposed to 5000 lux white LED light for 3 h. For light exposure, the rats were kept in the light box with LED light sources in six directions and a ring-shaped apparatus inside to allow rats to move one way in an anticlockwise direction (Figure 1). The distance between the right eye of the rat and the light source was kept relatively steady, and only one rat was exposed to intense light each time to avoid disturbance between rats. The light exposure experiment was performed between 6 PM and 8 AM the next day. The rats were intraperitoneally injected with HBS (5 ml/kg) 30 min before light exposure in the prevention group or 5 days after light exposure in the treatment group. In the light damage group, medical saline was injected (5 ml/kg per day for 5 days, after intensive light exposure) instead of HRS. After light exposure, the rats were returned to the dim cyclic light environment. Five days later, electroretinography (ERG) recordings were obtained and retinal morphology was observed.

### ERG test

ERGs were recorded by using procedures previously described [23]. In brief, after 10-h dark adaptation, the rats were intraperitoneally anesthetized with ketamine (120 mg/kg body weight) and xylazine (120 mg/kg body weight). Their pupils were then dilated by using 0.5% tropicamide. ERGs in both eyes were recorded off of the corneal surface using a silver-chloride electrode loop encased in a layer of 1% methylcellulose. Stainless steel needle electrodes that had been placed in the cheek and tail acted as reference and ground leads, respectively. Full-field (Ganzfeld) stimulation was used to record ERGs by using a commercial system (RETI port; Roland Consult GmbH, Brandenburg, Germany) with a band pass of 0.5–1000 Hz. During ERG recording, scotopic conditions of 0.01 cd.s.m^-2^ ERG, 3.0 cd.s.m^-2^ ERG, and 3.0 cd.s.m^-2^ Ops (oscillatory potentials) were sequential recorded at first and then photopic ERGs of 3.0 cd.s.m^-2^ ERG and 3.0 cd.s.m^-2^ Flicker ERG were recorded after 10-min light adaptation under 30 cd.m^-2^. The amplitude and/or latency of ERGs were analyzed.

### Measurement of retinal outer nuclear layer (ONL) thickness

In order to determine the relationship between retinal structure and function after light exposure and the role of HRS in retinal protection in rats, the thickness of the ONL was measured by morphometry across the vertical meridian in stained sections of the retina. After ERG testing, animals were euthanatized by an overdose of carbon dioxide, and the eyes were enucleated, fixed, and embedded in paraffin. The obtained retinal sections were stained with hematoxylin and eosin (H&E). Care was taken to avoid edema in making the tissue sections. Sections (5 μm thick) were taken along the vertical meridian to allow for comparison of all regions of the retina in the superior and inferior hemispheres [24]. For each section, digitized images of the entire retina were captured using a digital imaging system (DP71, Olympus, Japan) at 4 × magnification with 1360 × 1024 pixels. In each hemisphere, the ONL thickness was measured at 0.5, 1, 1.5, 2, 2.5, 3.0, 3.5, and 4.0 mm starting at the optic nerve head and extending along the vertical meridian toward the superior and inferior ora serrata.

### Statistical analysis

All data were expressed as mean ± standard error. Comparisons between groups were carried out using one-way ANOVA with Tukey’s multiple comparison tests. P < 0.05 was considered statistically significant. Data reported in the Results section are representative of at least three independent experiments.

## Results

### Functional evaluation by ERG

**Figure 2:**
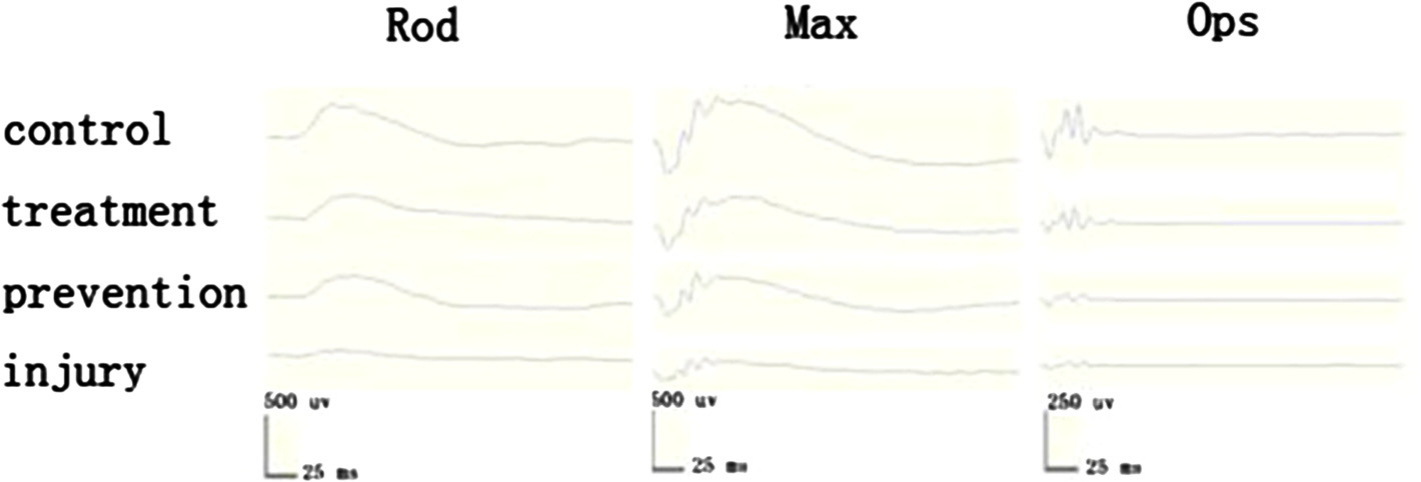
**Responses for the dark-adapted conditions were detected at an intensity of 3.0 cd.s.m**^**-2**^**.** The ERG waves for the dark-adapted condition were detected (0.01 cd.s.m-2 ERG, 3.0 cd.s.m-2 ERG, and 3.0 cd.s.m-2 Ops ). Scotopoic ERGs responses were decreased larger in light damage than prevention and treatment groups compared with blank control.

ERGs of all rats were recorded at 5 days after acute light exposure. The effect of intense light on the retina was compared between unexposed and exposed eyes, and a significant difference was found. The decreased ERG amplitude (scotopoic and photopic 3.0 cd.s.m^-2^ ERG) was greater in the light damage group than in the prevention and treatment groups (Table 1), especially the waves of scotopoic (3.0 cd.s.m^-2^) ERG (Figure 2). There was about 70% or more loss of ERGs amplitude in light damage group (*P* < 0.001) compared with the blank control group, 50% loss in hydrogen-rich saline prevent group, and 30% loss in hydrogen-rich saline treatment group (*P *< 0.001), respectively.

**Table 1:** Functional data recorded from four groups by ERG

	**n**	**Rod**	**Max**	**Ops/μV**	**cone**	**flick/μV**
		b/μV*	b/μV*	**	b/μV*	*
Blank control	6	385.00 ± 67.86	581.33 ± 55.30	152.67 ± 65.15	81.75 ± 14.33	39.55 ± 12.38
Light damage group	6	99.55 ± 49.96	166.83 ± 11.87	45.27 ± 10.58	17.53 ± 3.90	9.87 ± 4.58
Prevention group	6	198.83 ± 34.20	276.00 ± 89.83	84.43 ± 21.42	39.55 ± 12.98	23.92 ± 4.56
Treatment group	6	294.50 ± 62.77	394.50 ± 56.61	114.97 ± 36.57	53.07 ± 13.92	25.37 ± 9.19
*F*		29.704	52.198	8.106	29.717	12.652
*P*		0.000	0.000	0.001	0.000	0.000

Data are expressed as means ± SD. The changes of electroretinograph components in SD rats that were administrated with HRS before light exposure (prevention group) and after light-induced damage for the following 5 days (treatment group) were recorded from right eyes of each rat, while the control group data came from unexposed eyes. *significant among the four groups, P < 0.001, **P < 0.01.

### Morphological evaluation by quantitative histology

**Figure 3:**
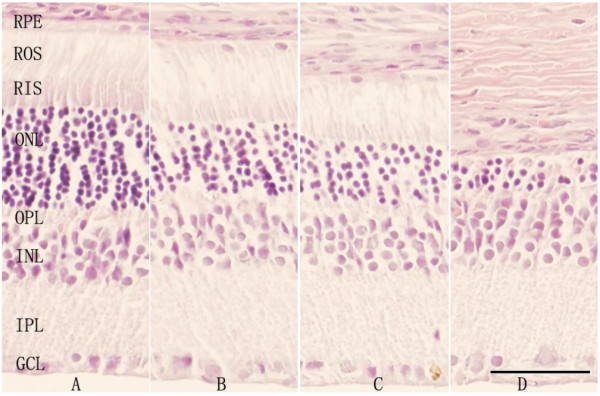
**Representative histology of the superior retina (measured at 1–2 mm starting at the optic nerve head) in age P56 SD rats, 5 days after light exposure.** The unexposed retina is shown in panel **A**, and the retina that was injected with HRS after light exposure is shown in panel **B**. The retina that was injected with HRS before light exposure is shown in panel **C**, and the light-exposed retina is shown in panel **D**. Scale bar, 50 μm.

The retina of unexposed SD rats (blank control group) was normal, and layers of organized structure were clear (Figure 3A). In the light damage group (Figure 3D), the retinal pigment epithelium (RPE) was intact, but there was a remarkable loss of RPE cell nuclei in exposed eyes. The whole retina of rats in the treatment group was almost normal except the ONL was a bit thinner than the unexposed control (Figure 3B), while the ONL thickness in the prevention group was between those of the light damage and treatment groups (Figure 3C).

**Figure 4:**
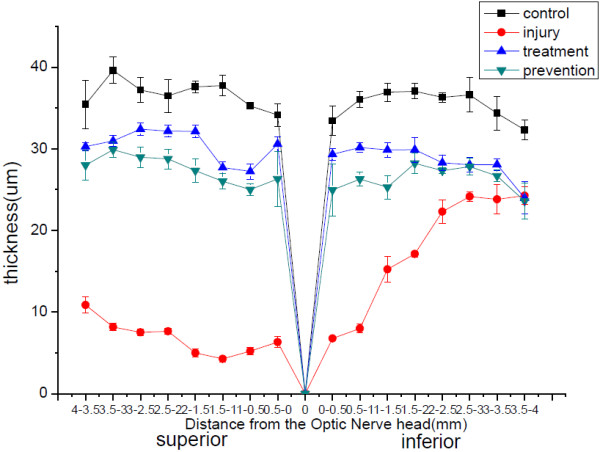
**Morphometric analysis across the vertical meridians of retinas in SD rats reared in dim cyclic light.** Black lines: unexposed to intense light; red lines: rats exposed at P56 and then maintained in darkness for 5 days; blue lines: HRS was given after light exposure; green lines: after HRS was given for 30 min, rats were exposed to light. The sample size was six rats per condition, with six ONL measurements per data point.

Figure 4 shows the ONL thickness in unexposed eyes and the eyes of the light damage, HRS prevention, and HRS treatment groups using morphometry across the vertical meridian. There were significant differences of two groups comparison between the damage group and the other groups(*P < 0.001*). HRS showed significant protective effect both injected to the rats either before or after the light exposure. In the light damage group, there was a large loss of photoreceptors over most of the superior half and the central portion of the inferior half. In the prevention group, the reduction of ONL thickness was restricted to the middle of the superior retina (*P <0.01,* compared with blank control), which previously has been identified as a sensitive region [24]. In the treatment group, did not decrease significantly after exposure to light for 5 days HRS injection, especially the sensitive region, but it did not achieve normal levels (*P < 0.01,* compared with blank control).

## Discussion

The retina is one of the most metabolically active tissues in the body and is sensitive to a wide range of ambient light conditions. During the course of light exposure, the retina, especially the ONL that contains high concentrations of polyunsaturated fatty acids (PUFAs) in the membranes, can produce hydrogen-rich free radicals. There is a natural balance between oxidants and antioxidants to maintain normal retinal function and prevent free radical damage, but excessive free radical production during intensive light exposure is considered to play a major role in the pathogenesis of retinal light damage [7,8]. It has been widely reported that natural and synthetic antioxidants can prevent retinal light damage and photoreceptor cell loss [8]. However, whether injected hydrogen is effective in protecting the retina against light-induced damage is still unknown; in particular, *in vivo* evidence is lacking.

There is a growing body of evidence that hydrogen has a medicinal effect, although the molecular mechanism has yet to be elucidated. It has been reported that hydrogen has antioxidant and anti-apoptotic activities by selectively reducing cytotoxic oxygen radicals [15]. In this study, the subjects were exposed to intense light for 3 h, and the HRS was intraperitoneally administrated each day. There should be excessive generation of free radicals in rats during light exposure, while the amount and method of hydrogen administration might not be enough to support that hydrogen reacts directly with free radicals in a similar manner as natural antioxidants or antioxidant supplements. Hydrogen sulfide has been recognized as a signaling molecule as well as a cytoprotectant. Maybe it can protect against light-induced retinal damage by regulating Ca^2+^ levels [25], since it has been shown to have a therapeutic role in the retina. In the current study, we confirmed the protective role of HRS on light-induced injury in rat retinas. HRS administration both before and after acute light exposure significantly reduced retinal damage in rats and promoted recovery from injury. HRS showed both preventative and therapeutic roles on light-induced retinal degeneration. Leshun Zhou [26] reported that HRS treatment both before and after reperfusion also had beneficial effects against spinal cord ischemia-reperfusion injury which could be abolished by the mitoK_ATP_ channel blocker 5-HD. We speculated that the role of hydrogen might be as a single molecule more than as an antioxidant to protect against light-induced retinal damage. Hydrogen is a simple molecule, but it plays a therapeutic role in different animal model and human diseases. Thus, it is important to further explore the biological mechanism of hydrogen [27]. In addition, we demonstrated that HRS injection into the intraperitoneal cavity is a simple, easy, and effective method that can be readily adapted for potential clinical practice.

In summary, we provided further evidence that intraperitoneal HRS injection may effectively promote recovery from light-induced retinal injury. Injecting HRS may provide a simple, safe, effective, and low-cost method for clinicians to treat many oxidative stress-related diseases.

## Competing interests

There was no competing interests.

## Authors’ contributions

LT and LZ performed the majority of experiments and drafted the manuscript; FX provided vital recording tools and participated in making animal models; J An carried out the histological observation; YS participated in the Data Statistics; ZZ designed the study and was also involved in editing the manuscript. All authors read and approved the final manuscript.
